# A race to net zero—early lessons from healthcare's decarbonization marathon

**DOI:** 10.1093/haschl/qxad006

**Published:** 2023-06-20

**Authors:** Kyle Lakatos, Arianne Teherani, Sapna E Thottathil, Seema Gandhi, Sheri D Weiser, Claire D Brindis

**Affiliations:** School of Medicine, University of California, San Francisco, San Francisco, CA 94143, United States; Harvard Kennedy School of Government, Cambridge, MA 02138, United States; School of Medicine, University of California, San Francisco, San Francisco, CA 94143, United States; UC Center for Climate, Health, and Equity, University of California, CA, United States; UC Center for Climate, Health, and Equity, University of California, CA, United States; Department of Anesthesia and Perioperative Care, University of California, San Francisco, San Francisco, CA 94143, United States; University of California, San Francisco, San Francisco, CA 94143, United States; School of Medicine, University of California, San Francisco, San Francisco, CA 94143, United States; UC Center for Climate, Health, and Equity, University of California, CA, United States; UC Center for Climate, Health, and Equity, University of California, CA, United States; University of California, San Francisco, San Francisco, CA 94143, United States; Philip R. Lee Institute for Health Policy Studies, San Francisco, CA 94158, United States

**Keywords:** decarbonization, climate change, mitigation, net zero

## Abstract

Climate change poses a threat to healthcare systems; at the same time, healthcare systems contribute to a worsening climate. Climate-induced disasters are predicted to increase both the demand for healthcare services while also posing a threat to the integrity of healthcare systems' infrastructures and supply chains. Many healthcare organizations have taken initiatives to prepare for such disasters through implementing carbon emission–reduction practices and infrastructure reinforcement, through globally recognized frameworks and strategies known as Scopes 1, 2, and 3, and decarbonization. We explored the efforts of these early adopters to understand how they are thinking about and addressing climate change's impacts on healthcare. Through a process of reviewing the peer-reviewed literature, publicly available published documents, annual sustainability reports, conference presentations, and participation in a national decarbonization collaborative, we (1) provide a diverse set of examples showcasing the variety of ways healthcare systems are responding; (2) identify a set of emergent key themes to implementing decarbonization practices, such as the role of an organizational culture of iterative improvement and building systems of cross-organizational collaboration; and (3) synthesize the identifiable set of driving factors for long-term sustainability of these decarbonization efforts.

## Lay Summary

Climate change poses a threat to healthcare systems, while, at the same time, healthcare systems contribute to climate change. Disasters caused by climate change are expected to increase demand for healthcare services and affect healthcare infrastructure and supply chains. Some healthcare organizations have taken steps to prepare for these challenges by implementing carbon-reduction practices and infrastructure reinforcement. To do this, they are using globally recognized frameworks for reducing carbon emissions. Researchers studied these early adopters and their efforts to address climate change in healthcare. They reviewed a variety of published documents, annual sustainability reports, and conference presentations and identified key themes for implementing these carbon-reduction practices. They also identified driving factors for the long-term sustainability of these efforts. This research can help healthcare organizations better prepare for and address the challenges posed by climate change.

## Teaser

This article explores the impact of climate change on healthcare systems and the efforts of early adopters of decarbonization practices to address it. As climate-induced disasters are predicted to increase demand for healthcare services, some organizations have implemented a variety of infrastructure reinforcement and carbon-reduction strategies. Through review of the literature and participation in national decarbonization collaboratives, researchers identified key themes for implementing decarbonization efforts and driving factors for their long-term sustainability. This research can help healthcare organizations better prepare for and address the challenges posed by climate change.

## Healthcare's climate change dilemma

By 2030, climate change will add an estimated quarter-million deaths per year, with a concomitant increase of four billion dollars annually in direct damage costs related to health alone.^1^ As the frequency of climate-induced disasters rises, higher demands for healthcare services will also coincide with disruptions to healthcare supply chains and infrastructure (eg, simultaneous incidences of injury and illness from wildfires or flooding combined with road closures or evacuations).^2–5^ Magnifying this threat is the realization that acute damages to hospitals or manufacturing warehouses (eg, from fires, floods, or hurricanes) could quickly derail an entire supply chain and impact a wide net of healthcare systems. As a result, leaders in healthcare are faced with multitudinous challenges. To tackle these realities, regulatory and international agencies alike are calling upon healthcare systems to both reduce their carbon emissions and to prepare for current and future climate disasters.

The shift in narrative of healthcare's role in addressing climate change was largely amplified after a 2018 study found that 4.4% of total global greenhouse gas emissions (GHGs) were derived from the healthcare sector.^6^ Looking specifically at U.S. GHGs, that number near-doubles to 8.5%.^6^ Unfortunately, despite having double the emissions and the costs of healthcare expenditures per capita compared with other high-income countries, U.S. health outcomes have remained incommensurate.^6–10^ The methodology used to disaggregate and quantify the 8.5% of U.S. GHGs has currently only been applied to healthcare; however, for comparison, estimates provided by the U.S. Environmental Protection Agency show that the agriculture, transportation, and electric power generation sectors produce around 11%, 27%, and 25% of U.S. GHGs, respectively.^11^ And while the elimination of the 8.5% of U.S. GHGs from healthcare would not fully reduce the global effects of climate change, it is a promising first step and a direct intervention healthcare institutions can pursue to address public health disparities.^2–5^ Additionally, healthcare action on GHG reduction can be combined with both energy efficiency measures and emergency preparedness planning, contributing to long-term financial savings and the overall resiliency of healthcare institutions.

The political prioritization for global healthcare systems to reduce their GHG emissions gained momentum at the 2021 UN Climate Change Conference in Glasgow (COP26). The subsequent formation of the COP26 Health Program recognized the healthcare industry as one of the major contributors to global GHGs.^12^ Parallel to these international efforts, in April 2022, the U.S. Department of Health and Human Services (HHS), in partnership with the Biden-Harris Administration, followed the recommendations of the COP26 Health Program and issued a call to action to all U.S. federal health systems to (1) reduce their emissions by 50% by 2030 and achieve net zero emissions by 2050, (2) provide public reporting on their progress, (3) complete an inventory of supply chain emissions, and (4) develop climate resilience plans for their facilities.^13–15^ As of publication of this article, over 100 healthcare institutions, pharmaceutical companies, insurance companies, and professional medical associations have signed the White House/HHS “Health Sector Climate Pledge.”^16^ The Biden-Harris Administration is also advancing additional climate goals in the 2022 Inflation Reduction Act.

To inform the larger policy and practice dialogue on decarbonization of the healthcare sector, in this article we address how we think about reducing carbon emissions and why it is an important goal for the healthcare sector and compile a set of examples of early adopters and leaders in healthcare's decarbonization efforts. We then turn to identify the key emerging themes for implementing decarbonization practices, synthesize the driving factors for sustaining decarbonization efforts in healthcare, and recommend next steps to increase and sustain climate mitigation, adaptation, and resilience efforts across the healthcare sector.

## Achieving net zero through decarbonization

The Intergovernmental Panel on Climate Change (IPCC), the main international body that produces research and recommendations on climate change, defines “net zero” as industries removing all reliance on fossil fuels or getting as low as possible and offsetting the remainder of fossil-fuel based emissions.^17,18^ One proposed solution to offset industry GHGs is through purchased carbon credits. However, thus far, inconsistent data standards, varying definitions of carbon-credit quality, and a lack of market transparency have resulted in limited success according to some economists.^19^

Where net zero acts as the goal, a newer term, “decarbonization,” represents a broad array of activities that institutions can participate in to reduce their GHG emissions and mitigate their contributions to climate change.^20–24^ Alongside decarbonization strategies, “climate resiliency” approaches are aimed at anticipating, preparing for, and responding to climate change disturbances and disasters (also collectively referred to as “adaptation”). While mitigation practices represent most of the decarbonization efforts that are being adopted by health systems (eg, minimizing food and medical waste), adaptation practices also play an ancillary role in systems preparation (eg, infrastructure reinforcement and utilization of renewable energy sources).

Across the United States, the diversity of geopolitical driving factors (eg, economic reliance on fossil fuels, electrical grid capabilities, regulatory requirements, healthcare reimbursement models) limits a “one-size fits all” approach for decarbonization.^25^ Fortunately, multiple collaboratives, formed across public and private sectors, have shared their strategies and recommendations for best practices to reduce emissions.^22–28^ The most widely referenced framework sets forth to quantify, monitor, and track an institution's carbon footprint, known as Scopes 1, 2, and 3.^29,30^ Scopes 1, 2, and 3 represent the categorization of different sources of GHGs into three buckets: (1) direct emissions from owned or directly controlled sources, (2) indirect emissions from the generation of purchased energy (mostly electricity), and (3) all other indirect emissions that occur in producing and transporting goods and services (including the full supply chain). Of the total 8.5% of U.S. GHGs produced by healthcare, Scopes 1 and 2 together comprise only 18% of the emissions generated by a facility, leaving the remaining 82% under Scope 3.^10^ A summary of Scopes 1, 2, and 3, as well as examples of different metrics and strategies used in each is provided in Table 1.

**Table 1.** Definitions and examples of Scopes 1, 2, and 3; decarbonization strategies; and examples of healthcare systems pursuing these goals.

Scope	Definition (percent healthcare GHG emissions)	Examples of emissions sources	Strategies (decarbonization approach)	Healthcare system example
1	Direct emissions from owned or directly controlled sources, on site (7*%*)	On-site combustion (eg, central heating plant)	Conserve and optimize energy use (eg, reduce air changes overnight and weekends in unused operating rooms, establish lighting controls with timers and motion sensors) Transition to zero-carbon fuel sources Meet and exceed the current green building and retrofitting standards (eg, LEED) Fuel cell Solar panels	Kaiser Permanente Veterans’ Health Administration NHS England
		Facility-owned vehicles	Electrifying a transportation fleet	Department of Veteran Affairs
		Anesthetic gas	Manage anesthetic gas choices (eliminate desflurane) Use low rates for fresh gas flow Decommission or avoid construction of central nitrous oxide piping	University of California Health Providence Health Providence St. Vincent Medical Center
2	Indirect emissions from the generation of purchased energy, mostly electricity (11*%*)	Purchased electricity	Purchase renewable electricity Conserve and optimize energy use	Providence St. Vincent Medical Center University of California Health
3	All other indirect emissions that occur in producing and transporting goods and services, including the full supply chain (82*%*)	Pharmaceuticals and chemicals	Prevent disease exacerbation Launch appropriate use campaigns Maximize lower carbon alternatives for inhalers alternatives	NHS England Providence St. Vincent Medical Center
		Medical devices and supplies	Encourage resource stewardship Adopt/expand circular economy policies and practices related to reuse, reprocess, repair, repurpose, and recycle Adopt preferential purchasing with suppliers or providers that perform carbon disclosures	Yale (life-cycle assessment of single- vs. multi-use laryngoscope handles; not discussed here)^31^
		Food procurement	Offer more plant-based options Adopt food-waste-prevention programs	Sutter Health Valley Region University of California Health
		Other sources	Minimizing employee commuting (telework and telemedicine), air travel, and waste (clothing drives)	CommonSpirit Health (strategies not discussed in this paper)^32^

*Source:* Adapted from the Agency for Healthcare Research and Quality (AHRQ): B. Sampath et al., “Reducing Healthcare Carbon Emissions: A Primer on Measures and Actions for Healthcare Organizations to Mitigate Climate Change,” (prepared by the Institute for Healthcare Improvement under contract no. 75Q80122P00007), AHRQ Publication No. 22-M011, Rockville, MD: Agency for Healthcare Research and Quality; September 2022, https://www.ahrq.gov/sites/default/files/wysiwyg/healthsystemsresearch/decarbonization/decarbonization.pdf.

*Note*: GHG = greenhouse gas; LEED = Leadership in Energy and Environmental Design; NHS = National Health System.

## Examples of early adopters and leaders in healthcare's decarbonization

In Table 2, we provide a brief snapshot of some illustrative examples of healthcare systems that are adopting climate resiliency strategies and tackling the challenge of addressing Scopes 1, 2, and 3. Case examples represent a broad range of healthcare systems (eg, differences in geography, patient population, and payment models) currently leading the fight against climate change. These examples, by no means comprehensive in nature, do include efforts by the University of California Health system (UC Health), Providence, Kaiser, the Veterans Administration, CommonSpirit Health, and others.^23,32–40^ By including several examples of different types of strategies aimed at accomplishing the same decarbonization goal and scope, we encourage current and subsequent generations of leaders and their implementation teams to consider their own opportunities. Importantly, the table does not capture the necessary persistence and social will needed to respond to these complex undertakings.

**Table 2.** Examples of decarbonization efforts by healthcare organizations, scope, goals, and outcomes.

Initiative (or organization driving initiative)	Scope^a^ addressed and example	Goal of initiative	Brief description of initiative	Achievements of initiative or lessons learned from initiative
Kaiser Permanente (Kaiser Richmond Medical Center—50-bed tertiary care facility; part of larger system of care in multiple states	Scope 1. On-site combustion	Carbon neutrality achieved in 2020 for Scope 1 and 2 emissions and Select 3 emissions	Solar microgrid, a 250-kilowatt solar panel installed atop the center's five-level parking garage, connects renewable energy and battery storage to a pre-existing, diesel-fueled backup power system.	Microgrid supplements the hospital's electrical demand with cleaner energy, augmenting energy loads at peak hours, offsetting the need for power from the major grid, and reducing consumption by at least 365 000 kilowatt-hours annually, the equivalent of removing nearly sixty cars from the road per year.^35^
Veterans’ Health Administration (large integrated healthcare system in the United States, serving 9 million enrolled veterans each year)	Scope 1. On-site combustion	Pursue mission of care, while achieving energy efficiency and resilience identified through quadrennial energy audits	Through combined capital investment and third-party financing, VA upgrades its facility infrastructure and equipment while installing renewable power where feasible.	Has successfully reduced its energy intensity (kilo-British thermal unit (kBtu)/gross square foot) by over 26% since 2003. Sixteen VA hospitals overall use 38% less energy per square foot than the national average for all hospitals (which is approximately 234.3 kBtu/gross square foot.^37,41^
NHS England	Scope 1. Facility-owned vehicles	In 2021 transition to a fully zero-emissions ambulance fleet, aligned with the national specification to decarbonize, while ensuring the highest standards of safety and patient care	Purchases of zero-emissions ambulance fleet.	Decarbonizing the ambulance fleet is estimated to reduce emissions by 87 kilotons of carbon dioxide equivalents (ktCO2e).^27^
The Department of Veterans Affairs (VA)	Scope 1. Facility-owned vehicles	VA plans to transition its roughly 23 000 vehicle fleet to ZEVs	VA audits its medical facilities to assess and plan for charging infrastructure, while also acquiring solar chargers to provide more immediate capacity. Simultaneously, VA is replacing its petroleum-fueled vehicles with ZEVs.	Since October 2021, VA has confirmed 507 ZEV orders, which make up 34% of its light-duty vehicle acquisitions this annual cycle.^37^
UCSF	Scope 1. Anesthetic gas	To reduce emissions related to excessive volatile anesthetic consumption by lowering FGFs	Implemented an electronic health record–based clinical decision support tool within the Epic Anesthesia Information Management System (AIMS) aimed at reducing FGFs and evaluate the effectiveness of this intervention in achieving sustained reductions in FGF rates and volatile anesthetic consumption and cost.	Demonstrated a decrease in mean FGFs by 0.6 L/min (95% CI, 0.6–0.6 L/min; *P* < .0001) for sevoflurane and 0.2 L/min (95% CI, 0.2–0.3 L/min; *P* < .0001) for desflurane immediately after implementation of the intervention, resulting in decreased rates and cost per MAC hour.^33,34^
Providence Health and Services (8 regional hospitals in Providence, Oregon, including quartenary medical center)	Scope 1. Anesthetic gas	To reduce emissions associated with inhaled anesthetics	Used iterative clinical quality-improvement program, including personalized clinical benchmark reports and education.	Reduced inhaled anesthetic emissions by 4550 tons CO2e per year (equivalent to 980 passenger vehicles per year), largely through avoiding use of desflurane; sustained a 94% annual reduction in GHG emissions.^36^
Providence St. Vincent Medical Center	Scope 1.	To decommission central piped nitrous oxide and substituted portable E cylinders after discovering high rates of infrastructure leaks	The overall facility leak rate was 1.89 liters per minute (L/min), resulting in clinical use efficiency of 5.4%.	The decommissioning of central nitrous oxide piping reduced losses by 958 tons CO2e (the equivalent of 206 gasoline-powered passenger vehicles off the road) in one year and saved $12 000 USD in procurement costs. Similar assessments at twenty-four other Providence hospitals found leak rates ranging from less than 0.1 to more than 3.5 L/min, with use efficiency of 0.9 25%–29%, suggesting additional opportunity across the health system.^36^
UC Health system, including from UC's own electric company, UC Clean Power	Scope 2. Renewable electricity purchases	To obtain renewable energy from off-site sources, such as utility and establish UC's own self- provided electricity program (“UC Clean Power”)	UC Health is obtaining municipal retail tariff options and participating in UC's own electricity program.	Overall, 55% of UC's electricity use comes from renewable or carbon-free sources. UC Clean Power currently supplies approximately 40% of the University's purchased electricity, serving portions of nine campuses and five academic health centers that are eligible to participate. The program's portfolio includes power generated by two Fresno County solar projects under long-term contracts, as well as other renewable and carbon-free sources.^39^
NHS England	Scope 3. Pharmaceuticals and chemicals, inhalers	To reduce total GHG emissions from the NHS; and found that MDI prescriptions contribute approximately 3% of total GHG emissions from the NHS	In 2020, NHS England conducted a systemwide carbon footprint analysis that showed that the footprint of dry powder inhalers, for example, is approximately one-tenth that of MDIs, presenting a more sustainable alternative for prescriptions.	Reducing unnecessary prescriptions of MDIs is part of the NHS's strategy to achieve a net-zero carbon healthcare service.^27^
Providence Health and Services (8 regional hospital facilities, Oregon)	Scope 3. Pharmaceuticals and chemicals, inhalers	Reduce environmental costs, improve formulary decision making for inhaled medications	After performing a detailed formulary review of propellant-based GHG emissions for each inhaled medication formulation, Providence Oregon hospitals identified clinically equivalent MDI formulations of albuterol with 3-fold differences in emissions.	By prioritizing the lower emissions intensity inhalers, these emissions are projected to drop by 42%, or 298 tons of CO2e (the equivalent of 64 gasoline powered passenger vehicles driven) per year.
Sutter Health Valley Region (10 hospitals ranging in size from 40 to 523 beds; 1402 total beds)	Scope 3. Food procurement	To reduce wasted food	Northern California, USA. In 2020, Sutter Health implemented a food-donation pilot program in their Valley Region facilities to reduce wasted food. From January 2020 to February 2021, the 10 hospitals donated almost 65 000 pounds of food to more than 40 area nonprofits within 5 miles of each facility using a food-donation logistics company.	This program diverted food waste from the landfill, reducing carbon emissions by 142 metric tons CO2e (the equivalent of thirty-two gasoline-powered passenger vehicles driven for one year), and provided an estimated 55 000 meals to organizations addressing food insecurity in their communities. The pilot also led Sutter Health to expand the food donation program to six additional facilities within its health system.^23,40^
UCSF Health (3 hospitals with 1675 total beds)	Scope 3. Food procurement	Hospital sites that serve approximately 2.3 million meals annually, comprising 537 000 patient meals and 1.7 million retail transactions with faculty, staff, students, and visitors	UCSF Health joined the Cool Food Pledge aimed at achieving a collective target of reducing GHG emissions from food by 25% by 2030.	Between 2017 and 2020, UCSF Health's plant-forward efforts have reduced the climate impact of the food served by 12.5% overall, translating to an 8% reduction in GHG emissions per meal—equivalent to 455 gasoline-powered passenger cars off the road annually. This effort was driven by a reduction of beef procurement by 28%, an increase in legumes by 13%, and an increase of almost 70% in plant-based milk during this time period.^42^

*Note*: CI = confidence interval; FGF = fresh gas flow; GHG = greenhouse gas; MAC = minimum alveolar concentration; MDI = Metered dose inhalers; NHS = National Health System; UCSF = University of California, San Francisco; ZEV = zero emission vehicle.

a Scope 1 = direct emissions from owned or directly controlled sources, on site; Scope 2 = indirect emissions from the generation of purchased energy, mostly electricity; Scope 3 = all other direct emissions that occur in producing and transporting goods and services, including the full supply chain.

Taken as a whole, the examples offer opportunities for leaders in healthcare to learn about the feasibility and acceptability of these strategies. Activities listed are noted to both keep consistency with the organizational mission and address the global efforts of decarbonization, all without compromising on healthcare's goal to advance the value statement of patient care. While we highlight different types of examples (eg, building energy-efficient buildings, curbing flowrates on anesthetic gas, and refining hospital systems’ own vehicle fleets), Table 2 does not explicitly summarize systems that have pursued multiple strategies simultaneously. Furthermore, the table is not able to capture the layers of organizations’ accumulated experiences of testing and refinement. The examples are offered as illustrative, recognizing organizations’ ability to respond to their unique culture and set of resources. Rather than focusing on any one example or system, we provide a summary of key emergent themes for implementing decarbonization strategies and a set of five universal driving factors that are required to sustain them, regardless of strategy or setting.

## Key themes to implement decarbonization practices

While many of the healthcare systems reviewed pursued multi-scope strategies, it is important to recognize that even addressing Scope 1 activities (which are largely within the control of an organization) can present multiple challenges. For example, the established culture within an organization can be a major hurdle in a systemwide uptake of new decarbonization strategies. Yet, for others, a strong culture of iterative quality improvement has largely enabled organizations to make noteworthy progress in their decarbonization efforts. For example, individual hospital systems, such as University of California–San Francisco (UCSF) hospitals and Yale University Hospital, are utilizing electronic health records to change the type of and reduce the flow rate of anesthetic gases.^33,34^ On the national level, Practice GreenHealth (a nonprofit collaborative focused on developing environmentally conscious solutions for healthcare) facilitates multiorganizational collaborations in running pilots and scaling efforts.^38^ Additionally, many case examples also document clear financial incentives and outcomes (such as the cost savings cited by CommonSpirit and Kaiser).^32,35^ Importantly, a common thread seen across all examples is committed organizational leadership at both the executive and direct-provider level.

Creating widespread change to organizational policies and practices requires intentional and proven implementation methodologies. To ensure that progress towards decarbonization is effective, we found that institutions are utilizing several important tools developed for process improvement. One such example is Lean Six Sigma (LSS), a methodology embedded within a learning healthcare system that uses data to drive quality improvement and reduce waste. LSS has been described in the literature and is composed of two process-improvement methods: lean and six sigma.^43^ LSS principles of waste reduction have helped many organizations advance their mission, meet their decarbonization goals, and simultaneously achieve a financial pay-off. Another tool developed by the Institute for Healthcare Improvement, the Framework for Strategic Improvement, adopts concepts of “will and idea gathering,” as well as providing additional support and strategies for process implementation.^44^ While not explicitly developed for the purpose of decarbonization, it has wide utility in mobilizing organizations across the country to pursue strategic and meaningful change, including initiatives on mitigating impact on climate change.

## Driving factors for sustaining decarbonization efforts

The field of climate change has benefitted from the multidecade efforts of scientists and advocates working across government agencies, as well as public and private organizations, to identify potential areas for improving environmental impact. Using a variety of data, analyses, strategies, and measurement milestones, the field has laid the groundwork for actions across a variety of major sectors that have the greatest likelihood of successfully impacting climate change. And, over time, they have provided increasing specificity, aiming to ensure that each sector can contribute to a global endeavor. A wide array of stakeholders, including international and national organizations, federal and state agencies, and networks of healthcare systems, have risen to the challenge of implementing a variety of strategies relevant to the decarbonization of the healthcare systems. We review some of the early lessons learned, as well as consider several future steps for extending this agenda to reach even greater numbers of systems and providers. Selection criteria for these driving factors emerged from the authors’ review of publicly available published documents, conference presentations, and peer-reviewed literature, and through participation in national decarbonization collaboratives.

### A critical role for multisectoral global, national, and state-driven policy and regulatory action

International frameworks for decarbonization offer important mapping guides but must also offer great flexibility in adapting to national and local resources, context, and structures. This has not been easy to establish, given the controversy surrounding climate change, with a historical lack of consistent government commitments and, often, little political will. Thankfully, multinational organizations have built upon these efforts to help create useful and pragmatic frameworks that map actionable implementation steps (eg, Scopes 1, 2, and 3). The most widely adopted GHG accounting system, the GHG Protocol, was released in 1998 by the World Business Council for Sustainable Development and the World Resources Institute.^45^ Building off the GHG Protocol, the National Health Service (NHS) in the United Kingdom launched the Carbon Management Programme in 2007. As an example of a national scale effort aimed at reducing GHG emissions and promoting sustainable practices, NHS has reduced emissions by over 30% and resulted in an estimated $200 million in savings.^24^

In the United States, governmental organizations (such as the HHS and the Department of Veterans Affairs [VA]) are all working towards climate change policies and strategies. Other stakeholders (such as the Joint Commission) are pursuing incentives and requirements aimed at reducing GHGs. For example, the VA's upgraded facility infrastructure and equipment reduced its overall energy intensity to 38% lower than the national average, while the Joint Commission is exploring regulatory polices around reducing the carbon contributions produced by healthcare, helping make healthcare organizations mitigate their effects on and become more resilient to climate change.^46^

What remains uncertain is the balance necessary to enhance and expand local creativity. Ultimately, governmental bodies must maintain flexibility while also expediting the implementation of meaningful, and consistently applied, standardized metrics and actions.^30^ While some early adopters have initiated their own independent strategies, increasingly, national learning collaboratives and partnerships (such as the National Academy of Medicine's [NAM’s] Action Collaborative on Decarbonizing the U.S. Health Sector) help provide platforms for organizations to learn best practices and offer peer support and healthy competition, thus enabling relevant sectors to advance complex agendas.^20^

Finally, state-level actors will also play a critical role in advancing this complex agenda. While progress is being made in Scope 1 and 2 practices, it will undoubtedly require further government policy, regulatory, and financing incentives to advance Scope 3 actions, given its magnitude and the lack of internal organizational control. Whether through self-directed initiatives (eg, Michigan's recognition of the economic payoffs for engaging in climate change activities for sustainability), state regulatory incentives (eg, CA Climate Action Plan providing tax write-offs), or some combination thereof, states’ opt-in approach to climate change strategies, within and beyond the healthcare sector, will be essential towards reducing the nation's carbon emissions.^47,48^

### The importance of institutional leadership

Visionary healthcare organizational leaders represent a key ingredient needed to change systems. They establish organization-wide commitments, allocation of resources, and systemwide philosophies necessary to drive action. Many healthcare leaders are driven by the recognition that their organizational mission needs to be consistent with their strategies if they are to improve the lives of those they serve. Additionally, leaders of decarbonization efforts in healthcare organizations can now recognize the importance and track the efficacy of working across simultaneous scopes and strategies (ie, mitigation and adaptation).

To achieve ambitious climate goals, health systems are establishing and investing resources into creating Offices of Sustainability or designating visible individuals who drive the agenda (eg, Medical Director of Sustainability, of which the United States has only eleven). Many develop data dashboards that also help track climate change practices across their institution. For example, UC Health has worked to advance climate resiliency across their five academic medical centers through their Carbon Neutrality Initiative, adopting an implementation science lens that supports iterative learning cycles to reduce carbon emissions, as well as a dashboard to maintain accountability.^39,49^ Publicly available documentation of effective strategies and progress can provide a competitive edge in promotional marketing for those organizations that have played a leadership role. By implementing and testing a set of multipronged strategies, institutions can report upon a set of comparable metrics, learn from outcomes, and develop additional strategies. This speaks to showcasing and cultivating a culture of continuous quality improvement and learning.

### Educating and engaging clinicians to be advocates

Clinicians represent an important driving factor for healthcare's decarbonization.^5^ As the first line of healthcare delivery, their everyday actions in clinical decision making, through use of evidence-based practices and sharing of resources, can help direct organizations to greener practices. Physicians are motivated to promote the health and well-being of their patients and took the oath of “do no harm”; however, many have argued that their profession does in fact “do harm” through its impact on the environment.^50,51^

Education for sustainable healthcare refers to learning approaches that develop learners’ knowledge, skills, and attitudes about the interdependence of ecosystems and human health. This includes the effects of environmental change on health, the health sector's impact on the environment, and sustainable solutions to both problems.^52^ Sood and Teherani describe how an expanding health systems science trains clinicians (both current and the next generation) to think critically about health system function, resilience, and sustainability. This supplemental training also prepares the workforce to lead, innovate, and transform current health systems to prioritize planetary health, foster resource stewardship, and improve patient outcomes.^53^

As a result, educating clinicians about the environmental impact of healthcare, and the role they play in it, can help reduce the healthcare sector's carbon footprint (eg, such as avoiding unnecessary lab tests and imaging studies, encouraging telemedicine visits, advocating to reduce hospital energy consumption, preparing community members to respond to climate crisis). Developing a climate-focused workforce is one of the four core priority areas of both the Office of the Federal Chief Sustainability Officer (as underscored by HHS) and the NAM Action Collaborative on Decarbonizing the U.S. Health Sector.^20,54^ Preparing learners on how to practice *environmental accountability,* an idea that an institution's education, research, and service activities help to develop, promote, and protect environmentally sustainable solutions, can lead to a workforce more effectively addressing the health concerns of the communities they serve.^55^

### What gets measured gets done—building out implementation science in healthcare decarbonization

Across international and national organizations, there is a compelling commitment and focus on the role of metrics, dashboards, public reporting, and accountability (eg, GHG Protocol, Carbon Management Programme, Carbon Neutrality Initiative). This has contributed to advancing standardized ways for interorganization comparisons across different systems and strategies. For example, increasing cross-institutional awareness of the total GHGs from (and total spent on) goods and services, such UC Health's examination of food procurement, led to adopting food-waste prevention and diversion programs across multiple medical centers.^42^ While challenges still remain in selecting and implementing the right metrics, measures to monitor progress are key, including the analyses of why certain metrics were not reached and, positively, why they were.

By adopting recognized standards, early adopters have contributed to establishing evidence that implementing decarbonization practices is both feasible and doable. These accumulated experiences have helped build repositories of knowledge and best practices. This virtual bank of strategies provides a supportive framework for the next set of adopters that can be implemented within a broad range of contexts. It can also help inform global and national climate policy organizational drivers (eg, HHS's COP26 Health Program and NAM's Action Collaborative). It is this bidirectional approach that helps create the capacity necessary to overcome significant challenges faced by diverse organizations engaged in implementing climate change solutions. Thus, the healthcare sector (with its health equity focus) can be a learning platform and contribute to the decarbonization strategy repertoire for other industries.

While growing regulatory and other political incentives may pressure healthcare organizations to comply at a faster pace than initially faced by early adopters, the already accumulated knowledge provides an actionable roadmap for subsequent generations of decarbonization efforts. The field is fortunate to have its exemplars and reputable role models, across different systems and levels of healthcare, who have led the way in examining climate resiliency practices.

### The co-joining of healthcare decarbonization, health equity, and population health agendas to drive sustained change

Healthcare systems that commit themselves to decarbonization are driven by the recognition that they themselves are major contributors to the health status of their patients (eg, worsening air quality contributing to asthma, cancers, and heart disease). By implementing effective decarbonization strategies, healthcare systems can help ameliorate existing health disparities in part caused by climate change. Organizations, such as Providence, Kaiser, and CommonSpirit, among others, recognize climate change's cascading effects on health disparities, particularly among those already experiencing the greatest negative impacts (eg, reduction in lifespan). Responsive healthcare systems are prioritizing and growing their climate resiliency efforts to dually address the interwoven disparities between population and climate health. An encouraging outcome in this increasing pool of examples is the growing body of evidence demonstrating that, alongside their environmental and social cost savings, decarbonization practices can have concomitant economic benefits, all while maintaining a high standard of patient care and safety.^20,53,56–58^

A focus on the cross-calculation of the economic, environmental, and social costs, referred to as the triple bottom line, has been noted as the next phase to achieve healthcare value.^59,60^ Many organizations have already demonstrated economic savings with their decarbonization efforts, such as the VA's infrastructure and all-electric transport fleet investments.^37^ While a standardized form of cost comparison is still in the works, the Agency for Healthcare Research and Quality has put forward its recommendations for weighing different population health, environmental, and economic costs components.^23^ Additionally, economists are beginning to explore modeling methodologies, such as Dynamic Integrated Climate-Economy model (DICE), Policy Analysis of the Greenhouse Effect model (PAGE), and Framework for Uncertainty, Negotiation and Distribution model (FUND), to calculate the social costs of carbon (a carbon tax based upon social and environmental damages associated with GHGs in the future).^61^ These can be useful future tools for the healthcare sector to calculate their organization's and system-level environmental costs.

## Next steps

The synergistic work of global and national policy stakeholders, along with healthcare systems’ early adopters of decarbonization, have helped create scaffolding for implementing a variety of strategies across the healthcare sector. Future steps are needed to ensure that additional healthcare systems adopt a series of sustainable strategies that address all emissions in an ongoing manner. While many of the existent strategies reviewed here focus on Scope 1– and 2–related activities, these efforts help build the experience and confidence needed to embark on tackling new Scope 3 actions, which, while even more complex, present greater promise of an impact on decarbonization. Supportive system incentives are needed, including both regulatory (hospital accreditation) and financial (Centers for Medicare and Medicaid Services) approaches and health insurance reimbursement and taxes to propel a new series of strategies moving forward. Simultaneously, there is a need to encourage concurrent interventions that incorporate community voices to advance sustainable decarbonization.

Finally, it is naive to assume that healthcare will be able to complete its decarbonization efforts in isolation. So long as oil and fossil fuels act as the center of our energy sector, supply chain, and capital investments, healthcare will represent only one of the industries accelerating the harms of climate change. To achieve the major reductions needed in Scope 3 activities, it will be imperative for all sectors to collaborate and align the social, economic, political resources necessary to shift our production methods towards carbon-neutral and renewable energy sources. Although achieving net zero presently feels unnerving, we recall that a similar global effort was successfully undertaken to stop the harmful emissions of chlorofluorocarbon pollutants destroying the ozone layer.^62^ A powerful reminder that through a coordinated effort it is possible to change the tide of our international inertia.

## Supplementary Material

qxad006_Supplementary_Data
